# 

**Published:** 1857-10

**Authors:** 


September Index.

| | |
|---|---|
| National Hotel Disease | 197 |
| Law of Miasm and Malaria | 217, 199 |
| How to Lay in Coal | 202 |
| Going South | 206 |
| Patriarch's Letter | 208 |
| Who should Go West | 209 |
| Farming Errors | 211 |
| Parks of Cities | 212 |
| Care of Children | 214 |
| Our Country | 216 |
| Mortuary of New York | 218 |

October Index.

| | |
|---|---|
| " Inhalation" worthless | 232 |
| Economical Eating | 233 |
| Tea Drinking | 234 |
| " Hub Me" | 237 |
| Mental Power | 238 |
| Editorial Requisites | 239 |
| Bread, the best | 240 |
| Clerical Health | 240 |
| Milk of Cities | 241 |
| Gail Borden's Discovery | 242 |
| Travel by Rail | 244 |

